# Anæsthesia in Midwifery

**Published:** 1850-03

**Authors:** 


*Anæsthesia in Midwifery.*—We have of late heard but little of the exhibition of chloroform in labour through the public press, and it is, therefore, probable, that the interest attached to it is somewhat on the wane. If we may judge from the tenor of numerous private communications, there seems to be a growing indisposition, in this country, at least, to the employment of it in natural labour, though we are not prepared to say that the trouble attached to the use of the agent has not something to do with the objection. In instrumental and other forms of complicated labour, we believe that anæsthesia is still much employed by the leading accoucheurs in England. In Scotland, we understand that even in natural labour the withholding it is almost the exception. Dr. Simpson* has replied at length

*Philadelphia Med. Examiner, April, 1849.

to the objections urged by Dr. Meigs (vide Abstract, vol. ix., p. 318); but as he does not make use of any new arguments, we need not, having previously fully stated his view, go further into the matter.

Dr. Denham has also furnished a report on the use of chloroform; the main objects of which are succinctly stated in the following conclusions:

1st. Chloroform is a most valuable agent in all turning, forceps, and crotchet cases.

2d. In some cases of natural or difficult labour, it is useful in relieving pain, and relaxing the soft parts.

3d. If given in large quantities, or persevered in too long, it puts a stop to uterine contractions.

4th. In such cases the pains generally return with increased force, when the chloroform is discontinued.

5th. It does not relax the perineum more than the other soft parts.

6th. It may be administered so as to ensure immunity from pain, short of unconsciousness.—*Dublin Journal, from Ranking's Abstract. Report by Editor.*

